# The miRNA–mRNA regulatory networks of the response to NaHCO_3_ stress in industrial hemp (*Cannabis sativa* L.)

**DOI:** 10.1186/s12870-023-04463-w

**Published:** 2023-10-24

**Authors:** Kun Cao, Yufeng Sun, Xiaoyan Zhang, Yue Zhao, Jing Bian, Hao Zhu, Pan Wang, Baochang Gao, Xiaoli Sun, Ming Hu, Yongxia Guo, Xiaonan Wang

**Affiliations:** 1 Daqing Branch of Heilongjiang Academy of Sciences, Daqing, 163319 Heilongjiang China; 2 Heilongjiang BaYi Agricultural University, Daqing, 163319 Heilongjiang China; 3 National Coarse Cereal Engineering Research Center, Daqing, 163319 Heilongjiang China; 4 Heilongjaing Province Cultivating Collaborative Innovation Center for The Beidahuang Modern Agricultural Industry Technology, Daqing, 163319 Heilongjiang China

**Keywords:** NaHCO_3_, Industrial hemp, miRNA–mRNA regulatory networks

## Abstract

**Background:**

Industrial hemp is an important industrial crop and has strong resistance to saline-alkaline stress. However, research on the industrial hemp response to NaHCO_3_ stress is limited. Therefore, the response mechanisms of industrial hemp under NaHCO_3_ stress were analysed through miRNA–mRNA regulatory networks.

**Results:**

Seedlings of two salt–alkali tolerant and sensitive varieties were cultured in a solution containing 100 mM NaHCO_3_ and randomly sampled at 0, 6, 12, and 24 h. With prolonged NaHCO_3_ stress, the seedlings gradually withered, and the contents of jasmonic acid, lignin, trehalose, soluble protein, peroxidase, and superoxide dismutase in the roots increased significantly. The abscisic acid content decreased and then gradually increased. Overall, 18,215 mRNAs and 74 miRNAs were identified as differentially expressed under NaHCO_3_ stress. The network showed that 230 miRNA–mRNA interactions involved 16 miRNAs and 179 mRNAs, including some key hub novel mRNAs of these crucial pathways. Carbon metabolism, starch, sucrose metabolism, plant hormone signal transduction, and the spliceosome (SPL) were crucial pathways in industrial hemp's response to NaHCO_3_ stress.

**Conclusions:**

It is speculated that industrial hemp can regulate SPL pathway by upregulating miRNAs such as novel_miR_179 and novel_miR_75, thus affecting starch and sucrose metabolism, plant hormone signal transduction and carbon metabolism and improving key physiological indices such as jasmonic acid content, trehalose content, and peroxidase and superoxide dismutase activities under NaHCO_3_ stress.

**Supplementary Information:**

The online version contains supplementary material available at 10.1186/s12870-023-04463-w.

## Background

*Cannabis sativa* L., belonging to the family Cannabaceae, is used in industries at a tetrahydrocannabinol (THC) concentration of less than 0.3%, mainly for fibre, food, and medicine, and has great economic value [1, 2]. Heilongjiang province is one of the largest planting bases of industrial hemp. In 2017, local drug control laws were amended in Heilongjiang province, China, to regulate industrial hemp use [3].

Soil salinization is a critical factor limiting the normal growth and development of plants [4, 5]. The root and leaf surface areas and amount of dry matter mainly represent the plant response to salt-alkali stress [6]. Plants alter their osmotic regulatory substances and antioxidant enzyme activities to grow adequately under salt-alkali stress [5, 7, 8].

Salt–alkali stress alters the expression of related resistance genes involved in oxidative stress [9], osmotic regulation [10], hormone signal transduction [11], and ion homeostasis [12]. Seed germination is stimulated under a low concentration of neutral salt [2]. The saline-alkaline tolerance of various industrial hemp cultivars was compared owing to their different physiological indices [13]. Furthermore, some studies have revealed the molecular mechanisms of response to salt stress in industrial hemp [14, 15].

Highly conserved plant microRNAs (miRNAs) of approximately 22 nucleotides in length are small interfering RNA molecules that emerge as important gene expression regulators under stress [16, 17]. miRNAs regulate the gene transcription levels of target mRNAs in a sequence-specific manner [17, 18]. Additionally, miRNAs play an important role in enhancing plant tolerance against abiotic factors through negative or positive regulators approving amassing of positive regulators [19]. However, miRNAs and their molecular responses in industrial hemp under NaHCO_3_ stress have not been well studied. In this study, we aimed to identify differentially expressed genes (DEGs) and investigate the pathways associated with NaHCO_3_ stress. Seedling roots of 'Huoma No. 1' (salt-alkali-tolerant variety) and 'Jindao-15' (salt-alkali-sensitive variety) were used to perform miRNA–mRNA integrated analysis to explore the biological functions and the molecular mechanisms of industrial hemp response to NaHCO_3_ stress. In summary, these findings provide numerous candidate miRNAs and mRNAs for the innovation of industrial hemp salt-tolerant germplasm resources and the cultivation of saline-alkaline-tolerant varieties. Finally, this study may improve the resistance of industrial hemp, reduce the planting costs and increase the economic benefit.

## Methods

### Industrial hemp and NaHCO_3_ stress

Seeds of 'Huoma No. 1' (H) and 'Jindao-15' (J) were used as the experimental materials. The industrial hemp seeds were sterilized, germinated, and transplanted into lightproof boxes. The seedlings (three-leaf stage) were exposed to 100 mM NaHCO_3_. The root samples were randomly sampled under NaHCO_3_ stress for 0 h(H0, J0), 6 h(H6, J6), 12 h(H12, J12), and 24 h(H24, J24). Then, the samples were frozen in liquid nitrogen and stored at –80 °C. Each treatment was replicated three times [15]. The physiological indices of abscisic acid (ABA) and jasmonic acid (JA) were analysed using high-performance liquid chromatography (HPLC). The contents of lignin, trehalose, soluble protein, β-amylase, peroxidase (POD), and superoxide dismutase (SOD) were analysed using an enzyme-linked immunosorbent assay (ELISA). The assay kits were provided by Suzhou Mcy Bio-pharm Technology Co., Ltd. (Suzhou, China).

### Transcriptome analysis

Total RNA was extracted using TRIzol (Invitrogen, Carlsbad, CA, USA), and its concentration and integrity were determined using a Nanodrop 5000 (Thermo Fisher Scientific, Waltham, MA, USA) and Agilent 2100 (Agilent Technologies, Santa Clara, CA, USA). The miRNA was ligated to the 3′ SR and 5′ SR adaptors and reverse transcribed to create the first cDNA chain. Then, miRNA libraries were constructed using polymerase chain reaction (PCR) amplification and rubber cutting recycling of polyacrylamide gel electrophoresis (PAGE). Then, the constructed libraries were sequenced on an Illumina platform by Biomarker Technology [15]. DEGs were compared between J0 vs J12 and H0 vs H12. DEG analysis (mRNA with false discovery rate (FDR)< 0.01, fold change values ≥ 2; miRNA with P value≤0.05, fold change≥ 1.5), Gene Ontology (GO) team analysis (P value < 0.01), and Kyoto Encyclopedia of Genes and Genomes (KEGG) pathway analysis were conducted [20]. The protein‒protein interaction (PPI) network was analyzed using the STRING database (http://string-db.org/) and Cytoscape [15, 21, 22]. The original miRNA and mRNA sequencing data have been submitted to NCBI under accession numbers PRJNA 813212 and PRJNA 874321.

### miRNA target gene prediction and miRNA–mRNA integrated analysis

The miRNA target genes, derived from the DEGs in the industrial hemp samples, were predicted using TargetFinder software. Then, the miRNAs and their target genes were estimated, and an miRNA–mRNA integrated analysis was visualized using Cytoscape.

### Quantitative real-time PCR (qRT-PCR)

Ten mRNAs and two miRNAs were selected for qRT‒PCR to verify the reliability of RNA-seq. The qRT‒PCR primers are listed in Supplementary Table A2. The FastKing RT Kit (Tiangen Biotech Co., Ltd., Beijing, China) was used for reverse transcription, and Power qPCR PreMix(GeneCopoeia, Inc., Rockville, MD, USA) was used for qRT‒PCR. Each sample was normalized using *GAPDH* and *U6* as internal controls and performed in triplicate [15, 23].

## Results

### Morphological and physiological characteristics of industrial hemp seedlings under NaHCO_3_ stress

**Fig. 1:**
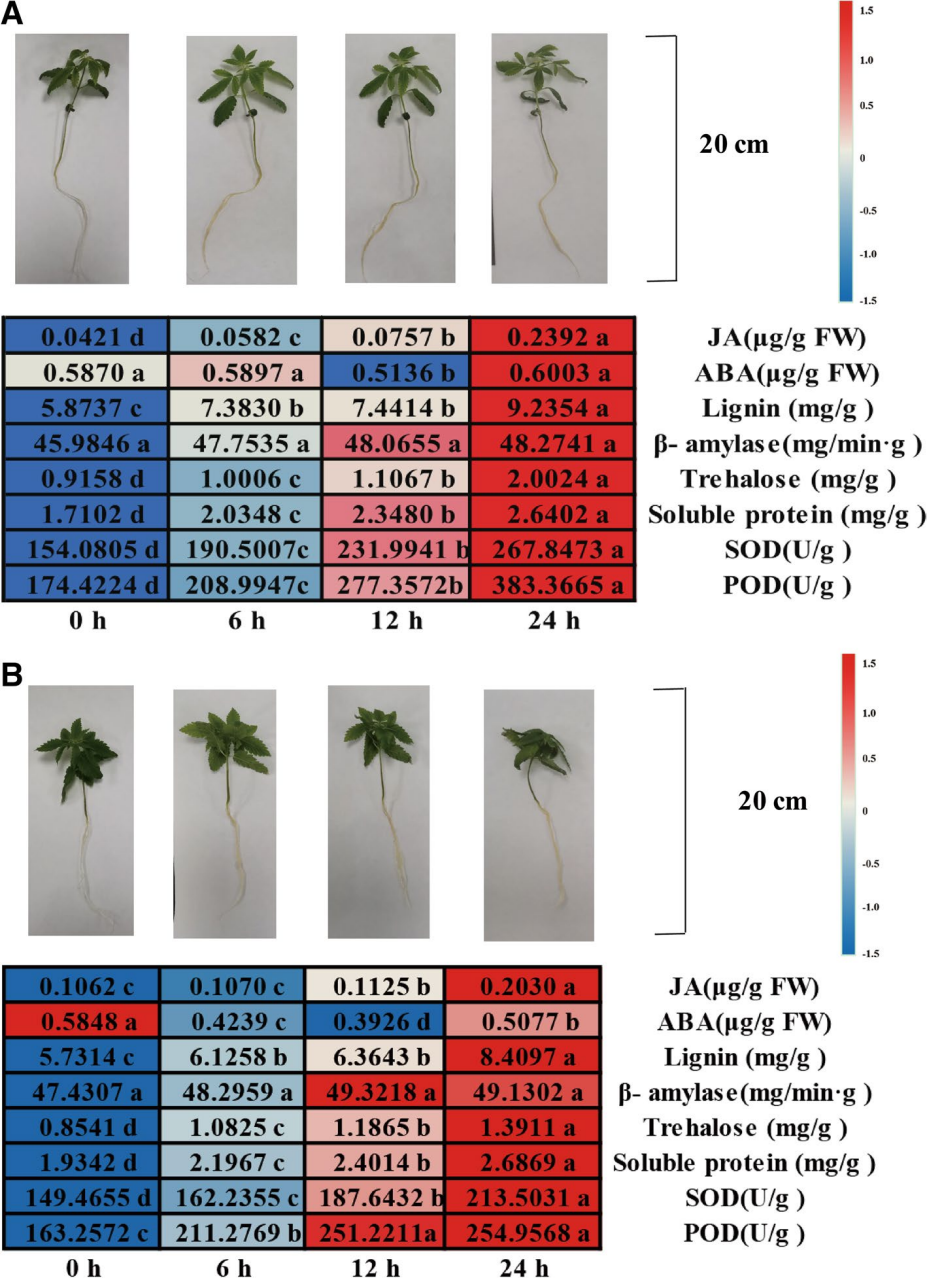
Morphological and physiochemical changes in industrial hemp under NaHCO_3_ stress. **A ** The morphological and physiochemical changes in Huoma No. 1 under NaHCO_3_ stress; values followed by different lowercase letters represent *p* ≤ 0.05. **B ** The morphological and physiochemical changes in Jindao-15 under NaHCO_3_ stress; values followed by different lowercase letters represent *p* ≤ 0.05

After 6 h of NaHCO_3_ stress, the industrial hemp seedlings grew adequately. However, the fibrous roots were slightly yellow in colour compared with those before stress. After 12 h, root yellowing became more severe. After 24 h, the seedlings were wilting, and the wilting of Jindao-15 (Fig. 1B) was more severe than that of Huoma No. 1 (Fig. 1A). The contents of JA, lignin, trehalose, soluble protein, POD, and SOD substantially increased with prolonged stress time. The content of β-amylase was not significantly increased, and that of ABA significantly decreased and then increased.

### Quality analysis of RNA-seq data

The 12 miRNA sequencing libraries were sequenced, and approximately 225.36 M clean reads were obtained. The Q-score at the Q30 level was more than 85%, and the ratio of tagged sequences compared with the known reference genome was between 8.25% and 30.45%. Among the 12 miRNA libraries, 290 miRNAs were obtained, including 4 known and 286 novel miRNAs (Supplementary Table A3). The 12 mRNA libraries were sequenced, and approximately 79.22 Gb clean data were obtained. The Q-score at the Q30 level was more than 93.71%, and the ratio of tagged sequences compared with the known reference genome was between 73.43% and 80.62% (Supplementary Table A4).

### DEGs under NaHCO_3_ stress

**Fig. 2:**
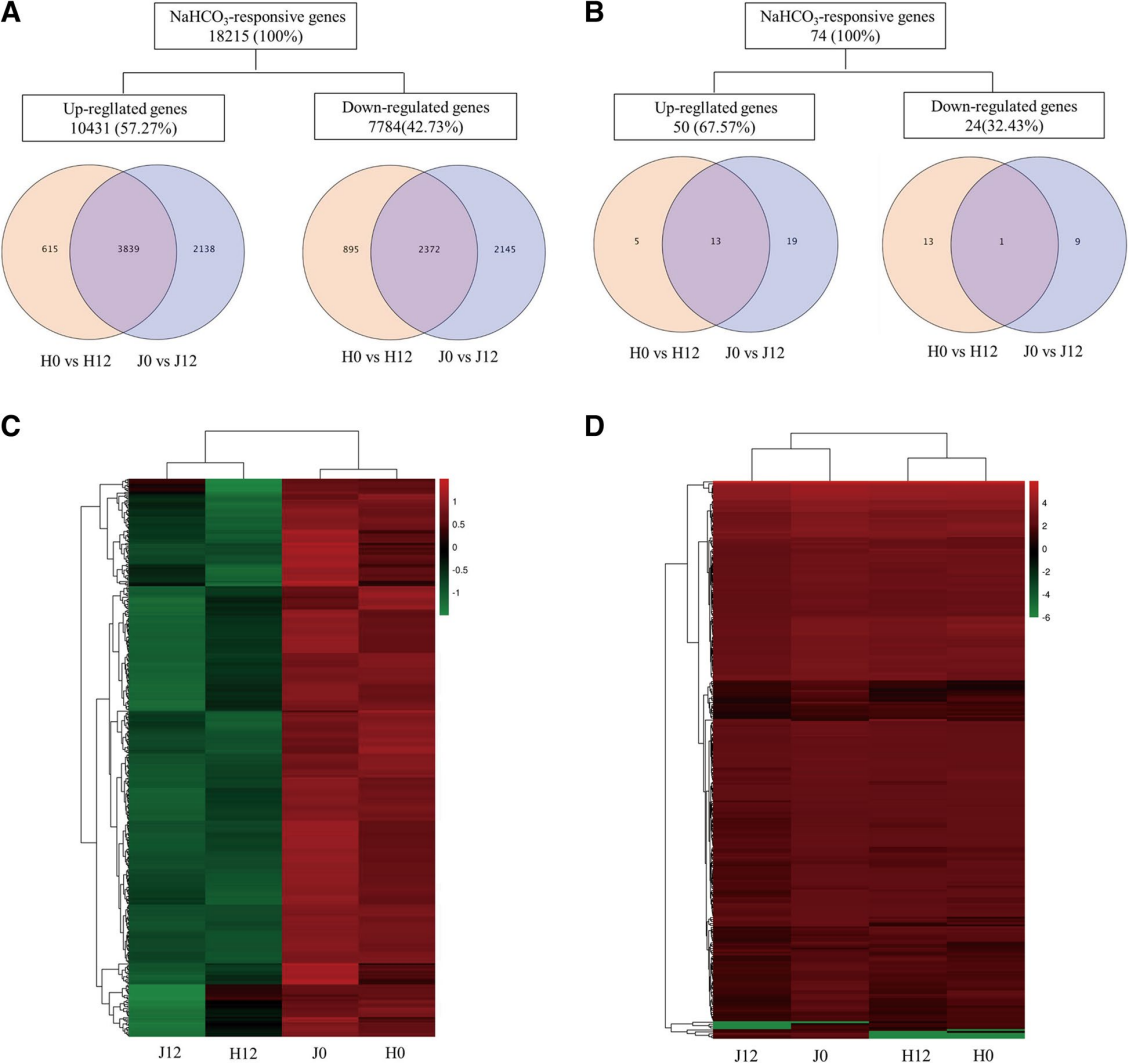
The expression profile of DEGs in industrial hemp roots under NaHCO_3_ stress. **A** Number of differentially expressed mRNAs. **B** Number of differentially expressed miRNAs. **C** Heatmap of mRNA expression profiles. **D** Heatmap of miRNA expression profiles

A total of 18,215 mRNAs (Fig. 2A) and 74 miRNAs (Fig. 2B) were identified as differentially expressed under NaHCO_3_ stress. Additionally, 57.27% of mRNA and 67.57% of miRNA were upregulated, and 42.73% of mRNA and 32.43% of miRNA were downregulated. The mRNA (Fig. 2C) and miRNA (Fig. 2D) expression levels were significantly different in response to NaHCO_3_ stress in the clustering heatmap.

**Fig. 3:**
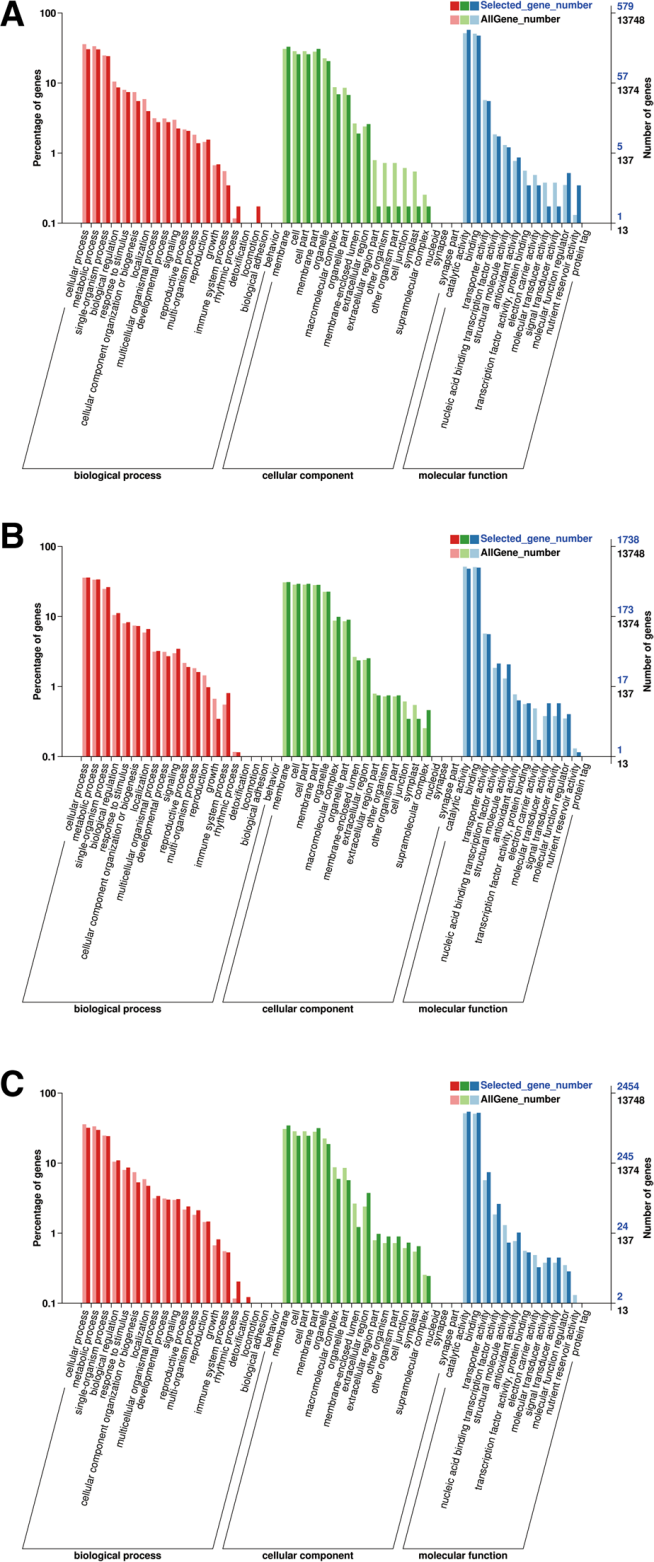
GO classification of the DEGs. **A** GO classification of DEGs in the (H0_vs_H12)_vs_(J0_vs_J12) group of Huoma No. 1. **B** GO classification of DEGs in the (H0_vs_H12)_vs_(J0_vs_J12) group of JinDao 15. **C** GO classification of co-expressed DEGs in the (H0_vs_H12)_vs_(J0_vs_J12) group

DEGs were classified by Venn diagram. The 1503 and 4267 differentially expressed mRNAs were assigned to 'Huoma No. 1' and 'Jindao-15', respectively. According to the GO enrichment analysis (Fig. 3), these DEGs were abundantly enriched in GO terms including cellular process (GO:0009987), metabolic process (GO:0008152), heterocyclic compound binding (GO:1901363), and organic cyclic compound binding (GO:0097159). The 6218 co-expressed DEGs were enriched in the membrane (GO:0016020), ion binding (GO:0043167), and cellular process (GO:0009987) GO terms.

The KEGG pathway enrichment analysis provided information on pathways of upregulated and downregulated DEGs under NaHCO_3_ stress (Supplementary materials_2). The upregulated DEGs in Huoma No. 1 were mainly enriched in the peroxisome, endocytosis, and carbon metabolism; however, the downregulated DEGs of Huoma No. 1 were mainly enriched in plant hormone signal transduction, the MAPK signalling pathway, and starch and sucrose metabolism. The upregulated DEGs of Jindao-15 were mainly enriched in plant hormone signal transduction, starch and sucrose metabolism, and protein processing in the endoplasmic reticulum. However, the downregulated DEGs of Jindao-15 were mainly enriched in the ribosome, carbon metabolism, and starch and sucrose metabolism. Furthermore, the upregulated co-expressed DEGs detected under NaHCO_3_ stress were mainly enriched in plant hormone signal transduction, the MAPK signalling pathway, and carbon metabolism. In contrast, the downregulated co-expressed DEGs were mainly enriched in plant hormone signal transduction, the MAPK signalling pathway, and starch and sucrose metabolism.

### Integrated miRNA–mRNA analysis

**Fig. 4:**
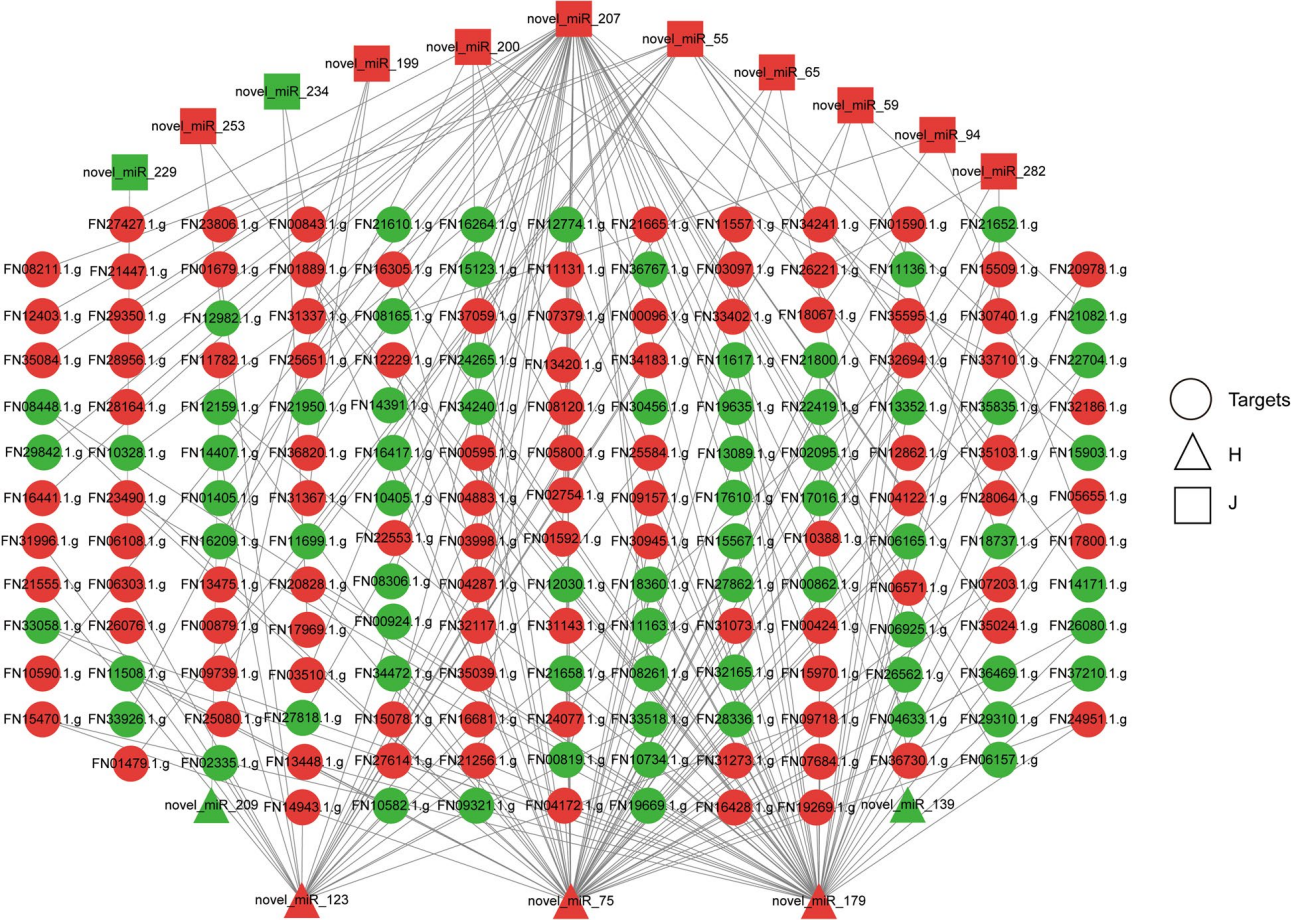
miRNA–mRNA interactions under NaHCO_3_ stress. The circles are target mRNAs. The triangles and squares are differentially expressed miRNAs of Huoma No. 1 and Jindao-15, respectively. Green and red represent downregulated and upregulated genes, respectively

**Fig. 5:**
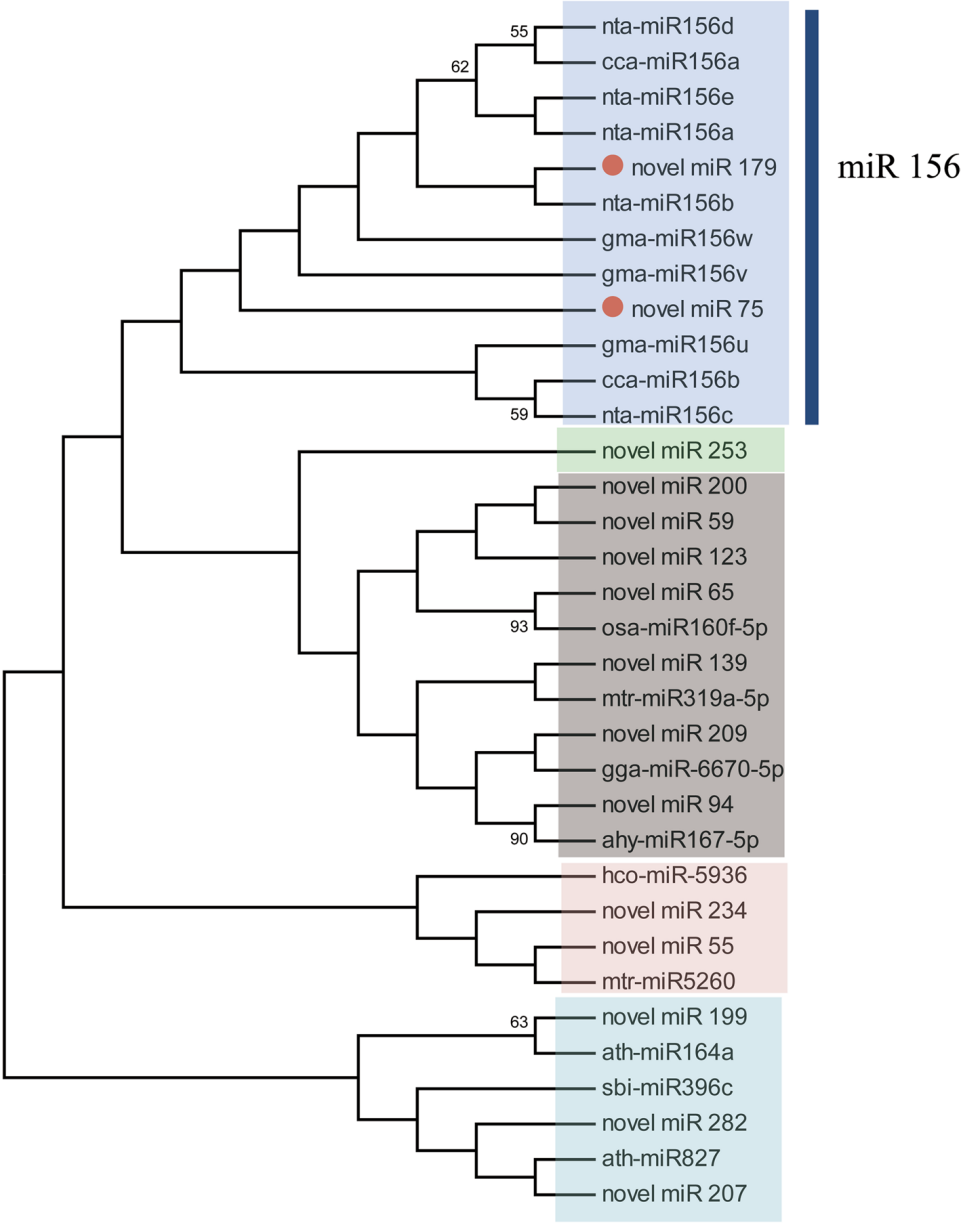
miRNA clustering using the neighbour-joining method.

A targeted regulatory relationship between miRNA and mRNA was visualized using Cytoscape. The network identified 230 miRNA–mRNA interactions involving 16 miRNAs and 179 mRNAs in Huoma No. 1 and Jindao-15 (Fig. 4, Supplementary materials_3). The neighbour-joining phylogenetic tree revealed that novel_miR_179 and novel_miR_75 clustered with cca-miR156 [24], nta-miR156 [25], and gma-miR156 [26] were clustered into one group (Fig. 5); novel_miR_207 and ath-miR827 [27] were clustered into another group; and novel_miR_55 and mtr-miR5260 [28]were clustered into a third group.

### qRT‒PCR validation of the RNA-seq data

**Fig. 6:**
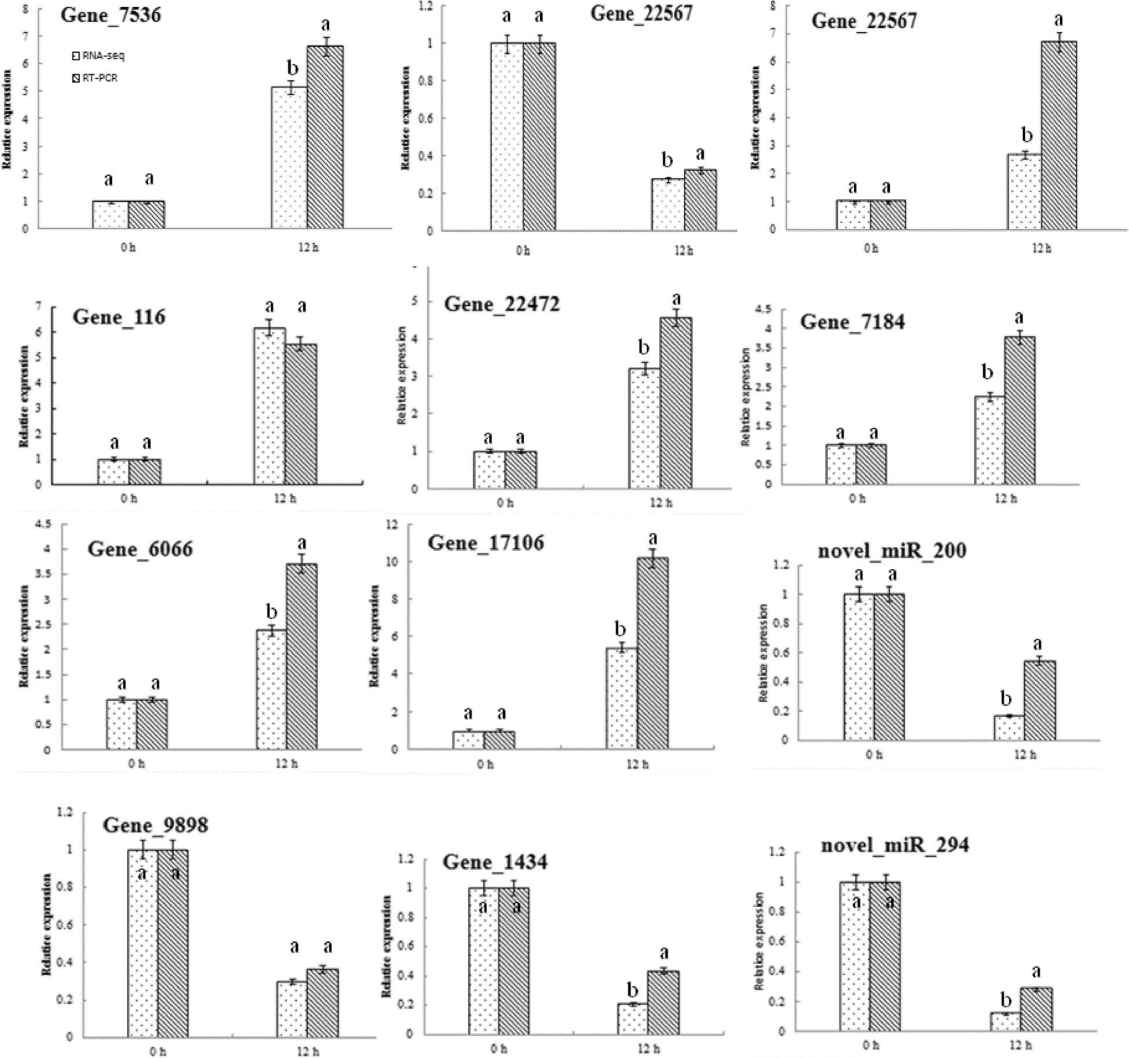
qRT-PCR analysis of differentially expressed miRNAs and mRNAs in industrial hemp roots under NaHCO_3_ stress

The expression levels of ten mRNAs and two miRNAs related to NaHCO_3_ stress were selected to validate the reliability of mRNA and miRNA through qRT-PCR. The qRT‒PCR results were similar to those of the RNA-seq analysis (Fig. 6).

## Discussion

The root morphology, traits, and physio-biochemical characteristics are generally affected by salinity stress [29–31], which hinders the normal growth, development, and biomass of the plant [5]. The inhibition of industrial hemp growth and development by salinity stress varies with the cultivars [32, 33]. This study used the morphology and physio-biochemical characteristics (the contents of phytohormone, lignin, trehalose, soluble protein, peroxidase, and superoxide dismutase) to describe the industrial hemp seedlings before and after NaHCO_3_ stress. Then, differentially expressed miRNAs and mRNAs in industrial hemp roots under NaHCO_3_ stress were analysed to explore the potential mechanism. Some significant miRNA-mRNA pairs and their potential roles were identified under NaHCO_3_ stress. Finally, the differences in physiological and biochemical characteristics before and after NaHCO_3_ stress in some important pathways were analysed. This paper offers a novel understanding of the response mechanism of other related species to alkali stress.

### RNA-seq analysis

Our study focused on the 10,431 upregulated and 7784 downregulated DEGs and the pathways of carbon metabolism, starch and sucrose metabolism, and plant hormone signal transduction. The enrichment of these pathways was consistent with the results of a previous study [15].

miRNAs respond to stress by regulating the gene transcription levels of target mRNAs [17, 18]. Several miRNAs related to salt stress tolerance have been found in *Nicotiana tabacum *[18], *Arabidopsis thaliana*[34], *Medicago truncatula *[35], *Oryza sativa *[36], and *Zea mays *[37]. In this study, a total of 18,215 mRNAs and 74 miRNAs were found to respond to NaHCO_3_ stress in industrial hemp. We identified significant regulation of the highly conserved miR156 family under NaHCO_3_ stress. miRNA156 expression increases salt stress tolerance and helps the plant withstand stress conditions until conditions become suitable [34, 38, 39]. The expression of the miR156 family (novel_miR_179 and novel_miR_75) was upregulated after 12 h of NaHCO_3_ treatment in the salt–alkali tolerant variety, indicating that NaHCO_3_ stress induced the expression of novel_miR_179 and novel_miR_75 and improved the adaptability of industrial hemp to the alkaline environment. The targeted regulatory relationship between mRNA and miRNA revealed that *NewGene_9378* (*PRPF 19*), FN20728.1.g (*PRPF 17*), and *NewGene_5122* (*DHX8/PRP22*) were regulated by novel_miR_179 and novel_miR_75, and these genes were annotated in the spliceosome pathway, which is mainly involved in the mRNA surveillance pathway, RNA transport, and spliceosome pathway [34]. Overexpression of HV-MIR827 improves stress tolerance in barley [27], and MTR-MIR5260 is related to the response of tobacco to biological stress [28]. In this study, novel_miR_207 and novel_miR_55 of the salt stress-sensitive variety were clustered with ATH-MIR827 and MTR-MIR5260, respectively. Hence, we speculated that novel_miR_207 and novel_miR_55 could also improve the salt adaptability of the salt stress-sensitive variety. Furthermore, the expression patterns of these miRNAs were different in the two varieties. Therefore, these differentially expressed miRNAs are helpful for the further study of the response and adaptation mechanism of different varieties of industrial hemp to salt–alkali stress [13, 40].

### Plant hormone signal transduction pathway

The regulatory role of hormones is essential under high salt stress [41]. The plant hormone signal transduction pathway revealed that the key hub genes (*newGene_13657* and *newGene_11233*) were related to proteins TIFY 6B and phosphatase 2C 8. TIFY positively responds to alkaline stress by the ectopic expression of *GsTIFY10a* and *AtTIFY10a* and *AtTIFY10b* knockout [42]. In this study, *newGene_13657* was upregulated, and JA content was also increased. After 12 h of NaHCO_3_ stress, *newGene_11233* was upregulated in the salt–alkali-sensitive variety but was normally expressed in the salt–alkali-tolerant variety. Under stress, the PP2C gene is upregulated and gradually downregulated in maize and Arabidopsis [43, 44], and its expression is negatively correlated with ABA signal transduction [45]. Herein, the ABA content decreased and then gradually increased, and the degree of decrease in the sensitive variety was higher than that in the tolerant variety. However, according to the variation in ABA content in industrial hemp, this gene would also be downregulated after a period of stress. Therefore, it is speculated that after NaHCO_3_ stress, the TIFY family-related genes were upregulated, and PP2C-related genes were upregulated and then gradually downregulated in industrial hemp roots, inhibiting ABA signalling in the short term, promoting jasmonate signal transduction, and ultimately regulating the strategies related to NaHCO_3_ stress.

### Starch and sucrose metabolism and carbon metabolism pathways

Starch and sucrose metabolism, a stress response, mediates plant responses to abiotic stresses [46, 47]. The starch and sucrose metabolism pathway revealed that the key hub genes *(newGene_2736* and *newGene_5139*) were related to glucan endo-1,3-β-glucosidase 1 and β-amylase 3 [48, 49]. The expression of the β-glucosidase and β-amylase-related genes was upregulated in industrial hemp roots after NaHCO_3_ stress. However, the expression of the β-glucosidase gene was normal in the salt–alkali-sensitive variety. The PPI of the carbon metabolism pathway revealed that the key hub gene (*newGene_1914*) was related to 3-hydroxybutyryl-CoA dehydrogenase[50]. The expression of *newGene_1914* was gradually upregulated in the salt–alkali-tolerant variety and downregulated in the sensitive variety. Relatively low starch content is typically characteristic of tissues undergoing rapid growth, and 3-hydroxybutyryl-CoA dehydrogenase maintains root meristem activity [50–52]. With prolonged NaHCO_3_ stress, β-amylase activity increased; especially 24 h after NaHCO_3_ stress, the β-amylase activity of the tolerant variety increased by 5.0%, and that of the sensitive variety increased by 3.6%. Previous studies have shown that the root biomass of the salt–alkali tolerant variety was greater than that of the sensitive variety under NaHCO_3_ stress, indicating that the difference in starch decomposability in the roots is responsible for the different salt tolerance of industrial hemp [13].

## Conclusions

**Fig. 7:**
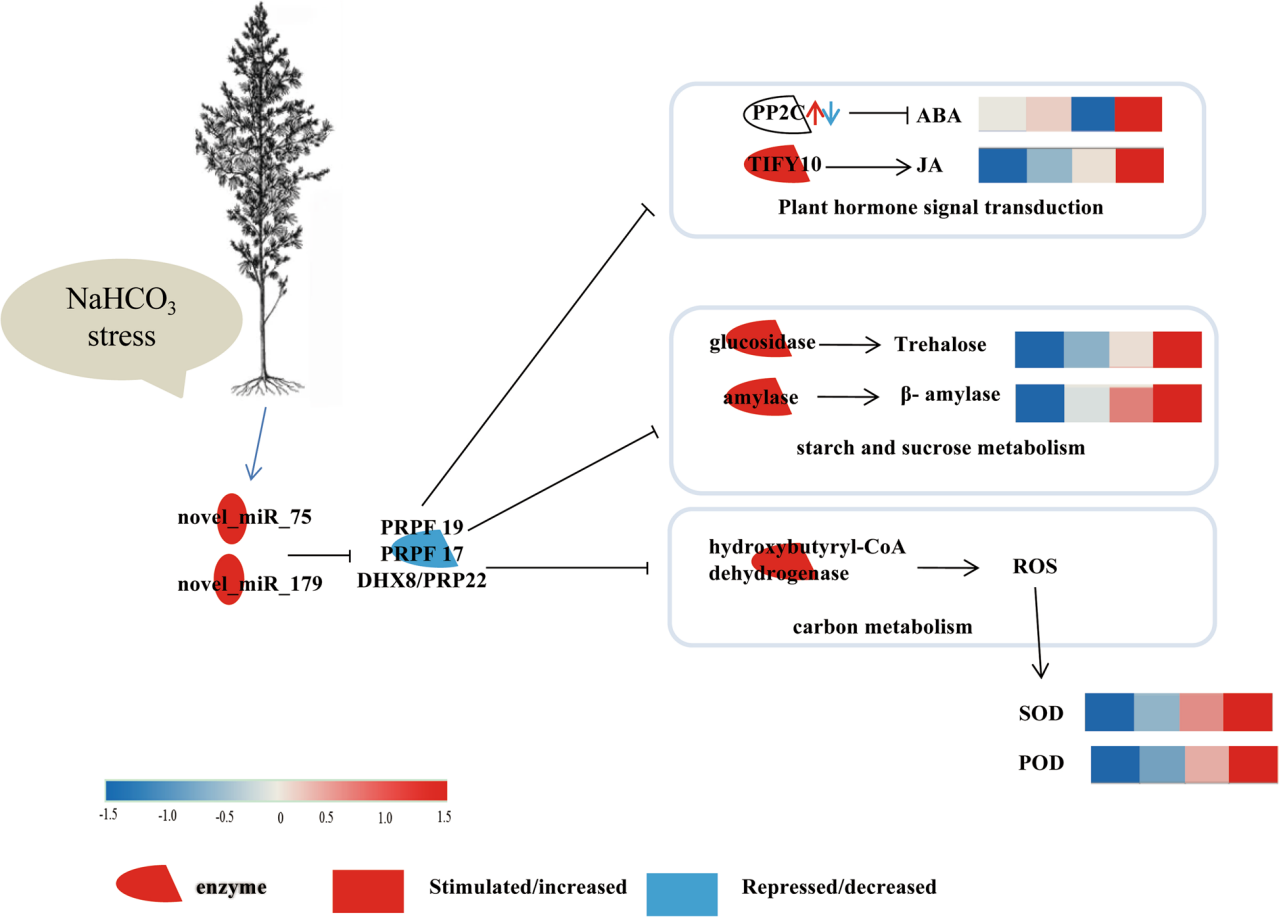
Important pathways in the response to NaHCO3 stress in industrial hemp

The miRNA and mRNA profiles of industrial hemp under NaHCO_3_ stress were comprehensively analysed. Overall, 18,215 mRNAs and 74 miRNAs were identified as differentially expressed in response to NaHCO_3_ stress. The common DEGs revealed that industrial hemp utilizes similar strategies, such as membrane, ion binding, and cellular processes, to respond to NaHCO_3_ stress. However, many essential biological processes, including endocytosis, plant hormone signal transduction, and starch and sucrose metabolism, were specifically enriched according to GO and KEGG pathway analysis under NaHCO_3_ stress. Starch and sucrose metabolism, carbon metabolism, plant hormone signal transduction, and the spliceosome were important pathways in the NaHCO_3_ stress response in industrial hemp. In addition, some novel miRNAs from the miR156 family with their target transcript genes were predicted as ideal candidates for future manipulation to improve NaHCO_3_ stress tolerance (Fig. 7). In summary, these findings provide a better understanding of the industrial hemp response to alkali stress at the miRNA and mRNA levels and provide candidate miRNAs and mRNAs for resistance breeding.

### Supplementary Information

**Additional file 1: Table A2.** List of primer sequences used in this study.

**Additional file 2: Table A3.** The quality of miRNA sequence data in three replicates for all samples.

**Additional file 3: Table A4.** The quality of mRNA sequence data in three replicates for all samples.

**Additional file 4: Supplementary materials_2.** The KEGG pathway enrichment analysis.

**Additional file 5: Supplementary materials_3.** The network of miRNA–mRNA interactions involving 16 miRNAs and 179 mRNAs among Huoma No. 1 and Jindao-15.

**Additional file 6: Table A1.** Quality of RNA samples used in this study

## Data Availability

The datasets generated and analysed during the current study are available in the NCBI repository, [ACCESSION NUMBER PRJNA 813212 and PRJNA 874321]”.
