# Supplementary material for: The miRNA–mRNA regulatory networks of the response to NaHCO3 stress in industrial hemp (Cannabis sativa L.)
Source: BMC Plant Biol. 2023 Oct 24;23:509. doi: 10.1186/s12870-023-04463-w (PMC10594861; doi:10.1186/s12870-023-04463-w)
Supplement: Supplementary file 3 — Additional file 3: Table A4. The quality of mRNA sequence data in three replicates for all samples. [file 12870_2023_4463_MOESM3_ESM.docx]

Supplementary Table A1. Quality of RNA samples used in this study

| Sample  (Replicate) | Concentration  (ng/ul) | Content  (μg) | 28S：18S | OD260/280 | OD260/230 | RIN*^a^* |
| --- | --- | --- | --- | --- | --- | --- |
| H0-1 | 201.2 | 3.0 | 2.03 | 2.12 | 0.5 | 8.7 |
| H0-2 | 260.5 | 3.9 | 1.98 | 2.13 | 1.07 | 8.5 |
| H0-3 | 353.7 | 5.3 | 2.04 | 2.13 | 1.78 | 8.5 |
| H12-1 | 278.4 | 4.2 | 2.07 | 2.12 | 1.21 | 8.2 |
| H12-2 | 221.2 | 3.3 | 1.97 | 2.09 | 1.63 | 8.4 |
| H12-3 | 205.3 | 3.1 | 1.83 | 2.1 | 0.9 | 8.2 |
| J0-1 | 187.8 | 2.1 | 1.79 | 2.15 | 1.45 | 9.3 |
| J0-2 | 203.2 | 3.0 | 1.84 | 2.11 | 0.5 | 8.6 |
| J0-3 | 214.3 | 3.2 | 1.83 | 2.11 | 1.56 | 8.5 |
| J12-1 | 218.4 | 3.3 | 1.99 | 2.1 | 0.66 | 7.6 |
| J12-2 | 200.3 | 3.0 | 2.14 | 2.12 | 0.61 | 7.7 |
| J12-3 | 214.8 | 3.2 | 2.13 | 2.1 | 1.93 | 7.6 |

*a*: RNA Integrity Number. RIN value ≥7.0 indicates a high quality RNA.
