# Supplementary material for: The miRNA–mRNA regulatory networks of the response to NaHCO3 stress in industrial hemp (Cannabis sativa L.)
Source: BMC Plant Biol. 2023 Oct 24;23:509. doi: 10.1186/s12870-023-04463-w (PMC10594861; doi:10.1186/s12870-023-04463-w)
Supplement: Supplementary file 4 — Additional file 4: Supplementary materials_2. The KEGG pathway enrichment analysis. [file 12870_2023_4463_MOESM4_ESM.docx]

Supplementary Table A2. List of primer sequences used in this study

| Gene name | gene-specific primers | | Probable encoded protein |
| --- | --- | --- | --- |
| *newGene_7184* | G7184_1F | GGTCCTATTGTCACCGAAGT | hypothetical protein F8388_003965 [Cannabis sativa] |
|  | G7184_1R | TAACTGCCTCATCCTGTCC |  |
| *newGene_**17106* | G17106_2F | GAATTGGTCCCTATGTTTGTG | beta-galactosidase-like [Cannabis sativa] |
|  | G17106_2R | CTTGAAAGGTCCATTGTCTGT |  |
| *newGene_6066* | G6066_1F | GGCTTTCAGTAGGGTTATCAGT | hypothetical protein G4B88_000932 [Cannabis sativa] |
|  | G6066_1R | TTGTTCAGGAGCTATGTATTTGA |  |
| *newGene_22472* | G22472_1F | TCGTAGGGCTGAAGAGAATAG | proline dehydrogenase 2, mitochondrial [Cannabis sativa] |
|  | G22472_1R | TATCAAGAGAGGAAGCAGACAA |  |
| *newGene_7536* | G7536_1F | ACAACAACAATAACCAGTCCTTT | B2 protein [Cannabis sativa] |
|  | G7536_1R | TCCTTCTTTCTCCAGTCCTTT |  |
| *newGene_116* | G116_2F | TTTGGTACAGGTGTTGGGA | uncharacterized protein LOC115722982 [Cannabis sativa] |
|  | G116_2R | CGTAGTTGAGGGTCTGCTTT |  |
| *newGene_9898* | G9898_1F | TGAGAAGGCAATCAAGGAA | enolase [Cannabis sativa] |
|  | G9898_1R | CGGAGAGATCAGCAATGAA |  |
| *newGene_1434* | G1434_2F | AAGAGATACCACAAGATGAAGGA | hypothetical protein F8388_025233 [Cannabis sativa] |
|  | G1434_2R | ATTGATAGCAGGGAACAACAA |  |
| *novel_miR_200* | m200_sl_1 | CCTGTTGTCTCCAGCCACAAAAGAGCACAATATTTCAGGAGACAACAGGGGAGCT |  |
|  | m200_1R | AGCGTTGGACTGAAGGG |  |
| *novel_miR_294* | m294_sl_1 | CCTGTTGTCTCCAGCCACAAAAGAGCACAATATTTCAGGAGACAACAGGTAGAGC |  |
|  | m294_1R | AGCGTTTGGATTGAAGGGA |  |
| CK1 | U6_1F | CAGAGAAGATTTGCATGGC | --- |
|  | U6_1R | TTTCTCGATTTGTGCGTGT |  |
| CK1 | GAPDH_2F | AATGAGCACGAATACAAGTCAG | --- |
|  | GAPDH_2R | AAGAGAATGAAATGAAATGAACC |  |
