# Supplementary material for: The miRNA–mRNA regulatory networks of the response to NaHCO3 stress in industrial hemp (Cannabis sativa L.)
Source: BMC Plant Biol. 2023 Oct 24;23:509. doi: 10.1186/s12870-023-04463-w (PMC10594861; doi:10.1186/s12870-023-04463-w)
Supplement: Supplementary file 5 — Additional file 5: Supplementary materials_3. The network of miRNA–mRNA interactions involving 16 miRNAs and 179 mRNAs among Huoma No. 1 and Jindao-15. [file 12870_2023_4463_MOESM5_ESM.docx]

Supplementary Table A3. The quality of miRNA sequence data in three replicates for all samples

| Samples | Total_Reads | Mapped_Reads | Raw_reads | Length<18 | Length>30 | Clean_reads | Q30(%) |
| --- | --- | --- | --- | --- | --- | --- | --- |
| H0-1 | 7172167 | 689835(9.62%) | 24985764 | 1024506 | 5106143 | 18855070 | 96.84 |
| H0-2 | 16881325 | 1776388(10.52%) | 55784313 | 1858984 | 11711074 | 42214197 | 98.24 |
| H0-3 | 21048979 | 1736255(8.25%) | 66610519 | 1808858 | 10272005 | 54529564 | 98.28 |
| H12-1 | 3257505 | 900479(27.64%) | 21279702 | 2998216 | 4378062 | 13903285 | 96.46 |
| H12-2 | 2523156 | 680106(26.95%) | 14574174 | 1954684 | 2107011 | 10512428 | 98.29 |
| H12-3 | 5724614 | 1357131(23.71%) | 19901435 | 1283480 | 3989899 | 14628017 | 98.40 |
| J0-1 | 9833761 | 1970224(20.04%) | 20910031 | 1160349 | 3104282 | 16645336 | 98.15 |
| J0-2 | 8864932 | 1811928(20.44%) | 17915740 | 1398222 | 1856595 | 14660882 | 98.08 |
| J0-3 | 6987193 | 1458963(20.88%) | 14082340 | 1584461 | 955155 | 11542659 | 98.28 |
| J12-1 | 4428382 | 971048(21.93%) | 16605461 | 2302140 | 2689964 | 11613304 | 98.21 |
| J12-2 | 3332892 | 894490(26.84%) | 16524788 | 4954310 | 1529572 | 10040759 | 96.49 |
| J12-3 | 1546852 | 470986(30.45%) | 12979466 | 4947288 | 1822523 | 6209540 | 98.26 |
