# Supplementary material for: The miRNA–mRNA regulatory networks of the response to NaHCO3 stress in industrial hemp (Cannabis sativa L.)
Source: BMC Plant Biol. 2023 Oct 24;23:509. doi: 10.1186/s12870-023-04463-w (PMC10594861; doi:10.1186/s12870-023-04463-w)
Supplement: Supplementary file 6 — Additional file 6: Table A1. Quality of RNA samples used in this study [file 12870_2023_4463_MOESM6_ESM.docx]

Supplementary Table A4. The quality of mRNA sequence data in three replicates for all samples

| Samples | Total Reads | Clean reads | %≥Q30 | mapped Reads |
| --- | --- | --- | --- | --- |
| H0-1 | 41184290 (100%) | 20,592,145 | 94.04% | 32106132 (77.96%) |
| H0-2 | 38499922 (100%) | 19,249,961 | 94.13% | 29935728 (77.76%) |
| H0-3 | 39174778 (100%) | 19,587,389 | 94.85% | 30461068 (77.76%) |
| H12-1 | 52918178 (100%) | 26,459,089 | 94.85% | 38858254 (73.43%) |
| H12-2 | 42326604 (100%) | 21,163,302 | 93.80% | 34042487 (80.43%) |
| H12-3 | 42491816 (100%) | 21,245,908 | 94.41% | 34255178 (80.62%) |
| J0-1 | 39342840 (100%) | 19,671,420 | 93.96% | 31333019 (79.64%) |
| J0-2 | 53646212 (100%) | 26,823,106 | 94.80% | 42588032 (79.39%) |
| J0-3 | 39448840 (100%) | 19,724,420 | 93.84% | 30939100 (78.43%) |
| J12-1 | 46657520 (100%) | 23,328,760 | 93.71% | 37111642 (79.54%) |
| J12-2 | 50238206 (100%) | 25,119,103 | 93.88% | 40067852 (79.76%) |
| J12-3 | 46703432 (100%) | 23,351,716 | 93.78% | 36833183 (78.87%) |
